# Invited Review: Factors associated with atypical brain development in preterm infants: insights from magnetic resonance imaging

**DOI:** 10.1111/nan.12589

**Published:** 2019-12-12

**Authors:** J. P. Boardman, S. J. Counsell

**Affiliations:** ^1^ MRC Centre for Reproductive Health University of Edinburgh Edinburgh UK; ^2^ Centre for the Developing Brain School of Imaging Sciences and Biomedical Engineering King's College London London UK

**Keywords:** brain, development, magnetic resonance imaging, neonate, preterm birth

## Abstract

Preterm birth (PTB) is a leading cause of neurodevelopmental and neurocognitive impairment in childhood and is closely associated with psychiatric disease. The biological and environmental factors that confer risk and resilience for healthy brain development and long‐term outcome after PTB are uncertain, which presents challenges for risk stratification and for the discovery and evaluation of neuroprotective strategies. Neonatal magnetic resonance imaging reveals a signature of PTB that includes dysconnectivity of neural networks and atypical development of cortical and deep grey matter structures. Here we provide a brief review of perinatal factors that are associated with the MRI signature of PTB. We consider maternal and foetal factors including chorioamnionitis, foetal growth restriction, socioeconomic deprivation and prenatal alcohol, drug and stress exposures; and neonatal factors including co‐morbidities of PTB, nutrition, pain and medication during neonatal intensive care and variation conferred by the genome/epigenome. Association studies offer the first insights into pathways to adversity and resilience after PTB. Future challenges are to analyse quantitative brain MRI data with collateral biological and environmental data in study designs that support causal inference, and ultimately to use the output of such analyses to stratify infants for clinical trials of therapies designed to improve outcome.

## Introduction

Preterm delivery, defined as birth at <37 weeks of gestation, is estimated to affect 10.6% of all live births around the world, which equates to 14.84 million births per annum 1. In resource rich settings, advances in perinatal care and service delivery have led to improved survival over the past two decades: around 25% of infants born at 22 weeks who are offered stabilization at birth will survive and this number increases to around 80% for births at 26 weeks 2. However, early exposure to extrauterine life can impact brain development and is closely associated with long‐term intellectual disability, cerebral palsy, autism spectrum disorder, attention deficit hyperactivity disorder, psychiatric disease and problems with language, behaviour and socioemotional functions 3.

## Computational magnetic resonance imaging of brain development after preterm birth

**Figure 1:**
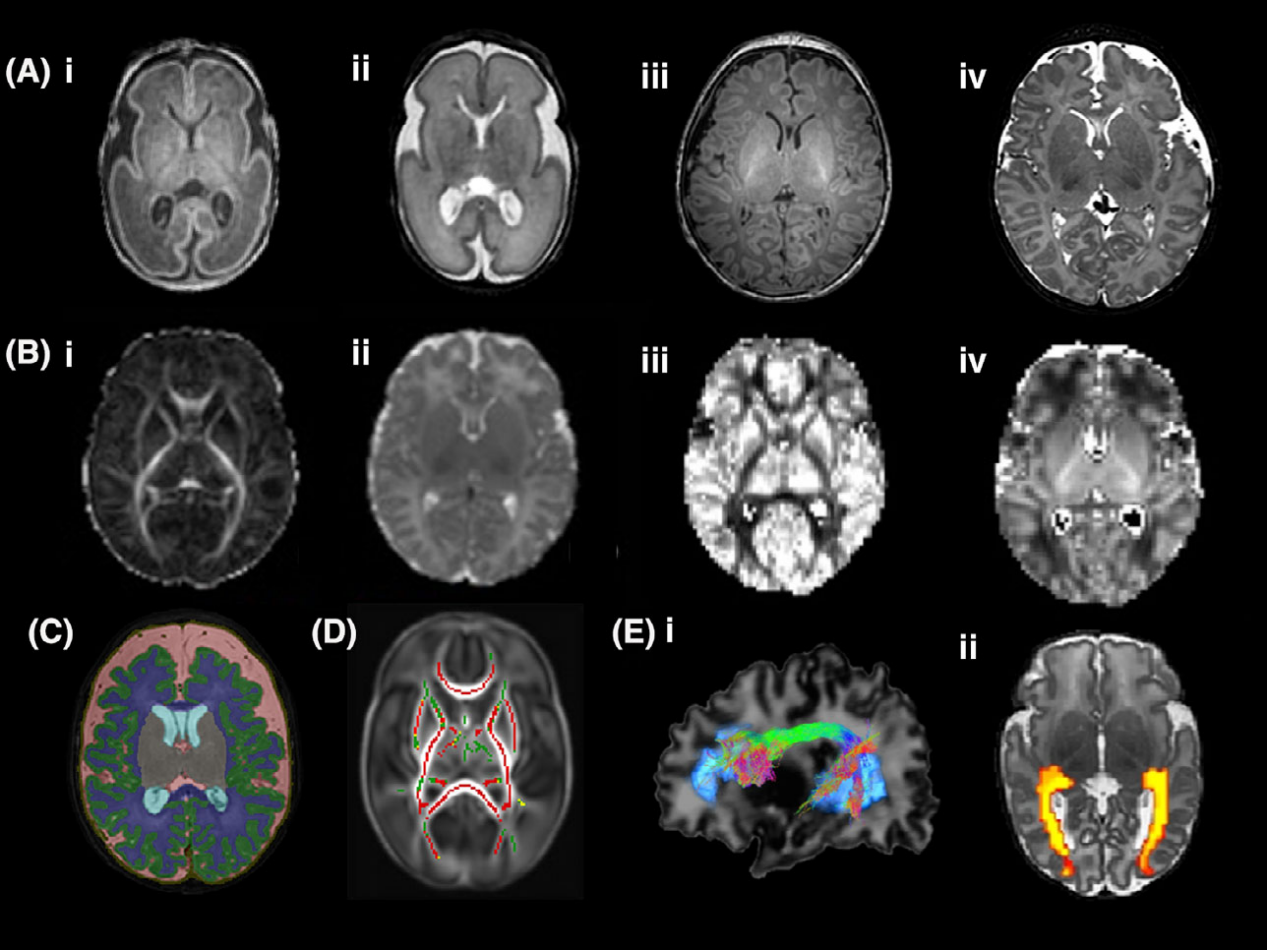
(**A**) (i) T1‐ and (ii) T2‐weighted images of an infant at 26 weeks gestational age (GA) and (iii) T1‐ and (iv) T2‐weighted images of an infant at 42 weeks GA at the level of the basal ganglia. (**B**) Diffusion magnetic resonance imaging maps at the level of the basal ganglia (i) fractional anisotropy, FA (ii) mean diffusivity, MD (iii) orientation dispersion index, ODI and (iv) neurite density index, NDI. (**C**) Brain segmentation in an infant born at 27^+4^ weeks gestational age and imaged at 41^+2^ weeks postmenstrual age. Key: Green = cortical grey matter, blue = white matter, grey = deep grey matter, pink = extracerebral cerebrospinal fluid. (**D**) Correlation between gestational age at birth and FA measures in white matter assessed using tract‐based spatial statistics. Mean FA skeleton (green) overlaid on mean FA map in the axial plane. Voxels showing a significant correlation (*P* < 0.05) between GA at birth FA are shown in red. (**E**) Diffusion MR tractography (i) arcuate fasciculus and (ii) optic radiations

The neuroimaging signature of preterm birth (PTB) includes alterations in white and grey matter microstructure, impaired cortical folding and disturbances to regional brain growth (Figure 1), for review see 4. Advances in foetal imaging enable direct comparisons between healthy foetal and preterm brain development at equivalent gestations and, although brain growth is rapid between 25 and 40 weeks of gestation in preterm infants 5, growth trajectories are slower in preterm infants than in healthy foetuses 6. At term equivalent age, regional brain volumes are reduced in preterm infants compared to healthy control infants and there is a reduction in cortical surface area, which may contribute to the neural basis of subsequent adverse neurodevelopmental outcome 5, 7, 8.

Diffusion‐weighted magnetic resonance imaging (dMRI) studies have provided valuable insights into the effects of maturation and injury on microstructural brain development. Biological inference from dMRI is rooted in the premise that water molecules move with Brownian motion in an environment without restrictions and change direction following collisions with other particles. In highly structured tissue such as brain, water movement is restricted by the presence of axons, neuronal cell bodies, glial cells and macromolecules, which supports inference about water content, axonal density, axonal calibre, myelination, dendritic arborization and synapse formation (for review see 9).

In general, anisotropy increases and mean diffusivity (MD) decreases with increasing maturation in the developing white matter of the preterm brain 10, 11 representing a combination of decreasing tissue water content and increasing complexity of white matter structures with age. Lower fractional anisotropy (FA) and increased MD are observed throughout the white matter in preterm infants compared with term‐born infants 12, 13 and increased prematurity is associated with lower FA and higher MD 14, 15. Diffusion tensor imaging metrics, such as FA, are nonspecific and reflect many underlying properties of brain tissue including neuronal density, fibre orientation dispersion, degree of myelination, free‐water content and axonal diameter. New approaches to analyse dMRI data, including those based on biophysical models such as neurite orientation dispersion and density imaging (NODDI) 16, are adding to our understanding of the preterm neuroimaging phenotype. The NODDI model aims to disentangle these different factors by separating the influence of neurite density and orientation dispersion from each other, to provide indices of orientation dispersion index (ODI), which captures the degree of dispersion of axonal fibre orientations (e.g. through fanning, bending, crossing) or dendrite orientations, and neurite density index (NDI), represented by the intracellular volume fraction 16. NDI increases with maturation in developing white matter and, at term equivalent age, NDI throughout the white matter is negatively associated with degree of prematurity at birth 17.

Unlike the changes observed in white matter, anisotropy and diffusivity in the developing cortical grey matter decrease with maturity and ODI increases reflecting dendritic growth from cell bodies, in‐growth of thalamocortical afferents, synapse formation and proliferation of glial cells 18. In comparison with term‐born infants, preterm infants at term‐equivalent age have increased cortical FA and cortical MD suggesting impaired cortical development 19, whereas lower gestational age (GA), lower birthweight and slower weight gain have been associated with higher FA in the preterm cortex 20.

## Perinatal factors associated with altered brain development in preterm infants

**Figure 2:**
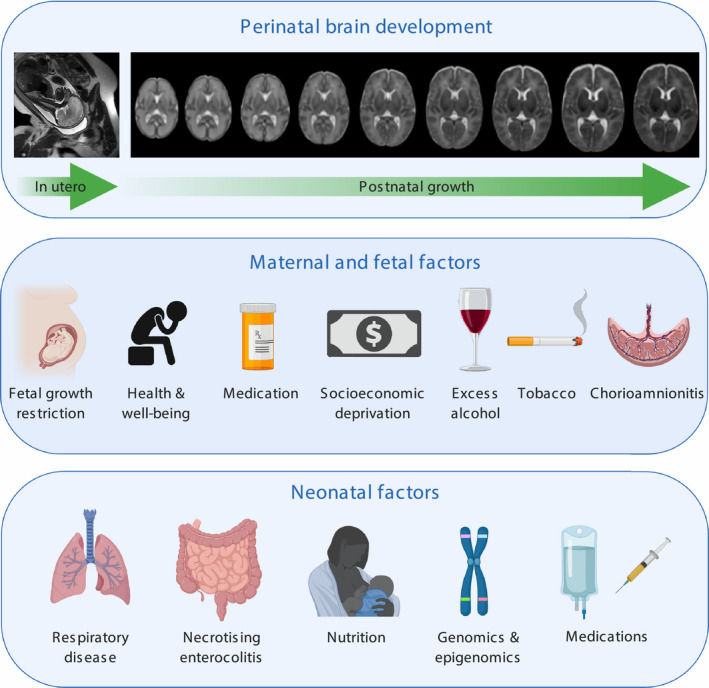
Maternal, foetal and neonatal factors associated with brain development in preterm infants

MRI of the brain in early life has opened opportunities to investigate maternal and infant factors associated with risk and resilience for healthy brain development (Figure 2).

### Maternal and foetal factors

#### Histologic chorioamnionitis

Chorioamnionitis is infection of the amniotic fluid, membranes, placenta and/or decidua, and it affects around 40–80% of very preterm deliveries. It can initiate a foetal inflammatory response that is injurious to the developing brain 21, and epidemiological evidence suggests an association between chorioamnionitis, cystic periventricular leukomalacia and cerebral palsy in preterm infants 22. We have shown histologically confirmed chorioamnionitis is associated with diffuse white matter disease at term equivalent age 23, although it does not appear to contribute to intraventricular haemorrhage or punctate white matter lesions on conventional imaging 24. This suggests that the pathway to atypical brain development begins *in utero* for some preterm infants.

#### Foetal growth restriction

Foetal growth restriction (FGR) refers to the foetus who does not achieve expected *in utero* growth potential due to genetic or environmental factors. FGR is closely associated with childhood sensory and motor deficits, cognitive impairment and cerebral palsy 25. MRI studies report atypical brain development in preterm infants affected by FGR, including reduced total and cortical grey matter volumes, reduced cortical complexity, reduced myelination, altered hippocampal and cerebellar development, changes in FA within the white matter skeleton and structural connectivity of specific brain networks 25. These data suggest that FGR preterm infants have a pattern of atypical development that is distinct from that seen in appropriately grown preterm infants.

#### Socioeconomic deprivation

Among the general population, brain tissue development and neurodevelopmental outcome are both patterned by socioeconomic gradients that operate in early life 26, and there is growing evidence that social disadvantage may exert additive risk to low GA for brain injury and impaired cognitive outcome in children born preterm 27, 28. Further work is required to understand the biological mechanisms that may link socioeconomic deprivation in the perinatal period with atypical brain development; plausible mechanisms include gestational immune dysregulation 29, alterations to the maternal hypothalamic‐pituitary adrenal axis 30, 31 and epigenomic variation associated with adversity in pregnancy 32, 33.

#### Maternal alcohol and drugs

Many studies report that prenatal alcohol exposure (PAE) is associated with atypical white matter in childhood, adolescence and adulthood 34, but studying the brain in later life introduces possible confounding by postnatal events and circumstances. MRI studies of neonates with PAE have reported altered dMRI parameters in white matter tracts, which suggests that atypical development is already established by the time of birth 35, 36. Maternal tobacco smoking is associated with lower global and regional foetal brain growth, after adjustment for somatic growth restriction 37.

Prenatal exposure to prescribed medications, specifically selective serotonin reuptake inhibitors, may influence neonatal brain structure and function 38, 39, including among preterm infants 40; and prenatal exposure to methadone for treatment of heroin addiction is associated with atypical white matter development 41. These observations from patients with depression and opioid use disorder raise urgent questions about the safety of maternal prescribed and nonprescribed drugs on the developing foetal brain. Neonatal MRI biomarkers may be useful for studies designed to disambiguate disease from treatment effects, and for investigating maternal pharmacotherapies that are safest for mother and foetus.

#### Maternal stress

An increasing body of evidence suggests that maternal prenatal stress exposure (PNSE) and anxiety/depression is associated with increased risk for a range of adverse behavioural outcomes in offspring including anxiety disorders 42, externalizing behaviour 43 and attention deficit hyperactivity disorder 44.

Recent studies provide evidence that the developing white matter is vulnerable to maternal prenatal adversity. Maternal anxiety is associated with reduced FA in key regions that are associated with anxiety, cognition and emotion regulation in later childhood including amygdala, cingulum, inferior temporal and frontal regions, angular gyrus, uncinate fasciculus, dorsolateral prefrontal cortex, cerebellum and inferior fronto‐occipital fasciculus, in term‐born infants 45. Dean and colleagues reported higher diffusivity and lower NDI in frontal white matter of term‐born infants of mothers experiencing prenatal symptoms of depression and anxiety 46, and we have observed higher diffusivity in the uncinate fasciculus in preterm infants at term equivalent age who experienced PNSE, even when controlling for GA at birth, socioeconomic status and the number of days on parenteral nutrition 47. Defining neonatal brain image markers of maternal stress offers new opportunities for investigating the biological pathways that link maternal well‐being with foetal brain development.

### Neonatal factors

#### Co‐morbidities of PTB

Bronchopulmonary dysplasia (BPD), defined as the need for supplemental oxygen and/or respiratory support after 36 weeks GA, complicates the postnatal course of around 30% of infants born with very low birth weight, and it is an independent predictor of poor neurodevelopmental outcome 48. Neonatal brain MRI studies of patients with severe respiratory morbidity, for example those with BPD or a requirement for prolonged mechanical ventilation, have reduced global and local brain volume 8, and reduced FA in white matter tracts 14 compared with age matched preterm infants without this complication.

Necrotizing enterocolitis (NEC; ischaemic necrosis of the intestinal mucosa) and blood stream infection in preterm infants often lead to a protracted systemic inflammatory response, and both are associated with neurodevelopmental impairment in early childhood. MRI studies suggest that severe NEC is associated with white matter injury, which might mediate the relationship between NEC and adverse neurodevelopmental outcome 49, 50, 51.

Retinopathy of prematurity is associated with reduced brain volume and altered white matter microstructure 52, 53, and the preterm infant, like the term infant, is susceptible to brain injury from bilirubin toxicity, hypocapnia and severe hypoglycaemia, so clinical policies designed to prevent these complications during neonatal intensive care are important.

#### Postnatal nutrition

Nutritional factors play an important role in preterm brain development and neuroimaging is a useful tool for investigating tissue effects of nutritional exposures. Optimal protein and energy intake in the first weeks after PTB are associated with increased brain growth, improved white matter microstructure and neurodevelopmental performance 54, 55, 56, and breast milk, as opposed to formula feed, during the weeks to discharge from Neonatal intensive care unit leads to improved structural connectivity of developing networks and greater FA in major white matter fasciculi 57.

#### Pain and medication

Very preterm infants are exposed to repeated painful stimuli as part of intensive care. The burden of painful exposures is associated with volume reduction in thalamic nuclei, altered thalamic metabolic function (decreased N‐acetylaspartate/Choline), reduced FA in thalamocortical networks and reduced functional connectivity, which implies that pain during this critical period of human development influences development of the somatosensory system 58, 59. Neonates who require intensive care sometimes require analgesic and/or sedative medications. Midazolam appears to have a dose‐dependent association with reduced hippocampal volume and microstructure, independent of procedural pain exposure burden 60. These studies raise important hypotheses about the possible roles of pain and medication in modifying preterm brain development, and they signal the MRI techniques that are likely to be most useful in future studies designed to evaluate the safety of medicines during neonatal intensive care.

#### Genomics and epigenomics

Imaging‐genomics methods are beginning to be used to investigate the contribution of genomic variation and epigenetic modifications to preterm brain development. For example single‐nucleotide polymorphisms at fatty acid desaturase 2, the 22q.11 locus, discs large MAGUK scaffold protein 4, and in the peroxisome proliferator‐activated receptor pathway are associated with altered FA in white matter, and polygenic risk for psychiatric disease is associated with abnormal deep grey matter development in preterm infants 61, 62, 63, 64. These early observations suggest that genetic variants may contribute to neuroanatomic variation after PTB, and that PTB might expose susceptibility to psychiatric disease.

DNA methylation (DNAm) provides a molecular link between early life stress and neuropsychiatric disease in adulthood. PTB is a profound physiological stressor that is associated with alterations in the methylome at sites that influence neural development and function, and exploratory analyses suggest that differential DNAm is associated with white matter development in preterm infants 32.

Integrated analysis of genomic data, differential DNAm and quantitative MRI offers new opportunity for understanding genetic and epigenetic bases of preterm brain injury, and the biological pathways that contribute to susceptibility and repair after PTB.

## Conclusions and future directions

MRI can be used to characterize brain development in terms of macro‐, and microstructure, function and metabolism. Combining features from neuroimaging with biological and/or clinical information has identified several maternal and neonatal factors that are associated with susceptibility to atypical brain development. Furthermore, analysis of data across different scales provides a framework for investigating whether and how determinants of brain development that operate in the general population such as maternal well‐being, drug exposures and socioeconomic gradients may interact with PTB to modify risk.

The observation that multiple types of exposure and genomic/epigenomic variants contribute to atypical brain development after PTB presents challenges for understanding causal pathways to injury and repair, and therefore for designing neuroprotective strategies targeted to the right infants at the right time. These challenges could be addressed by replication studies to assess generalizability, and by pooling image data from different centres to enhance study population sizes because scale‐up is needed to address issues of power and sensitivity, and to enable study designs that support causal inference.
